# Supplementary material for: Robust deep learning based protein sequence design using ProteinMPNN
Source: Science. Author manuscript; Available in PMC 2023 Mar 9. (PMC9997061; doi:10.1126/science.add2187)
Supplement: Supp Methods and S1-S9 [file NIHMS1874648-supplement-Supp_Methods_and_S1-S9.pdf]

Supplementary Materials for

Robust deep learning based protein sequence design using ProteinMPNN

J. Dauparas[1,2], I. Anishchenko[1,2], N. Bennett[1,2,4], H. Bai[1,2], R. J. Ragotte[1,2], L. F. Milles[1,2], B. I. M. Wicky[1,2], A. Courbet[1,2,3], R. J. de Haas[6], N. Bethel[1,2,3], P. J. Y. Leung[1,2,4], T. F. Huddy[1,2], S. Pellock[1,2], D. Tischer[1,2], F. Chan[1,2], B. Koepnick[1,2], H. Nguyen[1,2], A. Kang[1,2], B. Sankaran[5], A. K. Bera[1,2], N. P. King[1,2], D. Baker[1,2,3]*

[1] Department of Biochemistry, University of Washington, Seattle, WA, USA.
[2] Institute for Protein Design, University of Washington, Seattle, WA, USA.
[3] Howard Hughes Medical Institute, University of Washington, Seattle, WA, USA.
[4] Molecular Engineering Graduate Program, University of Washington, Seattle, WA, USA
[5] Berkeley Center for Structural Biology, Molecular Biophysics and Integrated Bioimaging, Lawrence Berkeley Laboratory, 1 Cyclotron Road, Berkeley, CA 94720, USA.
[6] Department of Physical Chemistry and Soft Matter, Wageningen University and Research, Wageningen, The Netherlands.

Correspondence to: dabaker@uw.edu

This PDF file includes:

Materials and Methods

Figs. S1 to S9

Materials and Methods

Methods for training single chain models

Training data

For single chain experiments presented in Table 1 we used a dataset based on the CATH 4.2 40% non-redundant set of proteins (*1, 7*). We trained models following the setup described in (*1*), i.e. using the learning rate schedule and initialization of the original Transformer paper (*20*), a dropout rate of 10% (*21*), a label smoothing rate of 10% (*22*), batch size with 6000 tokens, graph sparsity was set to be 30 nearest neighbors using Ca-Ca distances.

Architecture modifications

For experiment 1 we added extra input edge features, namely 16 Gaussian radial basis functions (RBFs) equally spaced from 0Å to 20Å for distances between N, Ca, C, O, and virtual Cb for i and j residues. This resulted in 25*16=400 edge features. The virtual Cb coordinates were calculated using ideal angle and bond length definitions: b = Ca - N, c = C - Ca, a = cross(b, c), Cb = -0.58273431*a + 0.56802827*b - 0.54067466*c + Ca.

For experiment 2 we introduced edge updates for the encoder network. The inputs to the encoder are node (denoted V_i) and edge (denoted E_{ij}) features for i and j residues. A message M_{ij} is constructed using a multilayer perceptron (MLP) applied to $[V_i, V_j, E_{ij}]$ concatenated tensors. These messages are summed over neighbors, j, and an additional MLP is applied to get a new updated nodes V_i^{new}. These new nodes are used to get new edges, $E_{ij}^{new}=MLP[V_i^{new}, V_j^{new}, E_{ij}]$. We used layer normalization, dropout, and residual connections for all layers, h_{new} = LayerNorm[h_{old} + Dropout(dh)], where dh is an output from the layer, h_{old} is the old value, h_{new} is the updated new value.

For experiment 4 we implemented a random decoding order when training. To achieve this we constructed a random permutation matrix on-the-fly for every input example and applied it to rows and columns of the upper triangular matrix which is an autoregressive mask for the left to right decoding. Alternatively, one could permute input tokens and keep the

autoregressive mask fixed given that the neural network architecture is permutation equivariant which is the case for most graph neural networks.

Methods for training multi chain models

Training data

We trained ProteinMPNN on protein assemblies in the PDB (as of Aug 02, 2021) determined by X-ray crystallography or cryoEM to better than 3.5Å resolution and with less than 10,000 residues. Sequences were clustered at 30% sequence identity cutoff using mmseqs2 (*10*) resulting in 25,361 clusters. We split those clusters randomly into three groups for training (23,358), validation (1,464), and testing (1,539), ensuring that none of the chains from the target chain or chains from the biounits of the target chain would be in the other two groups. Every training epoch, we cycled through the sequence clusters and picked a random sequence member from each cluster, and for each such 'query' we randomly picked a protein conformation (in cases where there were multiple) and reconstructed the biological assembly for the corresponding PDB entry; for cases with multiple biological assemblies, we picked one assembly at random. For hetero-oligomeric assemblies, we masked out the sequence from the query chain but provided the network with the sequence information on all other chains in the assembly, while for homo-oligomers, sequences were masked out from all copies to prevent potential information leakage (two protein chains were considered as homo-oligomeric if the sequence identity between residues aligned by TM-align (*23*) was higher than 70%.

Loss function and optimization

We used negative log likelihood with a label smoothing rate of 10% (*22*) for the loss (not using label smoothing works well too). The sum of negative probabilities worked much better than the average of log probabilities. The training loss was defined by $loss_{average}$ = sum(loss * mask) / 2000 where 2000 was chosen empirically, loss (categorical cross entropy per token) and mask had shapes [batch, protein length]. For optimization we used Adam with beta1 = 0.9, beta2 = 0.98, epsilon= 10−9, and the learning rate schedule described in (*20*). Models were trained using pytorch (*24*), batch size of 10k tokens, automatic mixed precision, and gradient checkpointing on a single NVIDIA A100 GPU. Training and validation losses (perplexities) as functions of optimizer steps are shown in Figure 3D. Validation loss converged after about 150k optimizer steps which is about 100 epochs of on-the-fly sampled training data from 23,358 PDB clusters.

Input features

ProteinMPNN input features were just embedded edges without any node features (Figure 1A). Using protein dihedral angles as node input features did not result in better performance so for simplicity in dealing with multichain backbones we did not use any node input features. The edge features consisted of distances between residues in Euclidean space and distances between residues in the primary sequence space (relative positional encoding) within a chain plus an indicator if residues are in different chains. We encoded distances between N, Ca, C, O, and virtual Cb (see Methods for training single chain models for the definition of Cb) for i and j residues using 16 RBFs equally spaced from 2Å to 22Å. For relative positional encoding we used AlphaFold (*9*) like discrete (one-hot encoded) tokens -32, -31,..., 31, 32 within the protein chains and additional token 33 if residues are in different chains. Ablating positional encodings showed almost the same performance suggesting that relative primary sequence or inter-chain information is already present in the Euclidean distances between atoms, e.g. distances between neighboring Ca atoms are the same for all residues.

Model architecture

We used encoder-decoder message passing neural networks for this task (*1*, *20*, *25*), see Figure 1. The encoder takes graph nodes and edges as inputs and using 3 layers with hidden dimension of 128 (larger hidden dimensions mainly decrease training loss with only marginal gains to the validation loss) updates those nodes and edges using message passing with edge updates.
Pseudocode for the encoder layer (V - node features, E - edge features):

```
def encoder_layer_forward(V, E):
    M_ij = MLP[V_i, V_j, E_ij]
    dV_i = Sum_j [M_ij]
    V_i = LayerNorm[V_i + Dropout(dV_i)]
    dV_i = FeedForward[V_i]
    V_i = LayerNorm[V_i + Dropout(dV_i)]
    dE_ij = MLP[V_i, V_j, E_ij]
    E_ij = LayerNorm[E_ij + Dropout(dE_ij)]
    return V, E
```

The decoder takes in node and edge features from the encoder plus encoded protein sequence to which an autoregressive mask is applied. The decoder layer is a vanilla MPNN layer with 3 layers and 128 hidden dimensions.
Pseudocode for the decoder layer (V - node features, E - edge features, S - sequence features, mask - autoregressive mask):

```
def decoder_layer_forward(V, E, S, mask):
    E_ij = Concat[E_ij, S_j] * mask_ij + Concat[E_ij, 0.0*S_j]  * (1-mask_ij)
    M_ij = MLP[V_i, V_j, E_ij]
    dV_i = Sum_j [M_ij]
    V_i = LayerNorm[V_i + Dropout(dV_i)]
    dV_i = FeedForward[V_i]
    V_i = LayerNorm[V_i + Dropout(dV_i)]
    return V
```

For both encoder and decoder the multi-layer perceptrons (MLPs) had 3 linear layers of the model's hidden dimension and GELU activation functions (GELU worked slightly better compared with ReLU). The FeedForward layers had 2 linear layers with GELU activations and with the middle hidden dimension 4 times bigger than the input dimension as in the Transformer paper (*20*).

Further in silico analysis

Amino acid compositional bias

Figure S2 shows ProteinMPNN and Rosetta amino acid compositional bias compared with the native PDB sequences. ProteinMPNN sequences were designed using temperature T=0.1. Rosetta designed sequences have an overrepresentation of alanines in the core and boundary. Both models favor a negatively charged glutamic acid, E, on the surface and disfavor a polar amino acid glutamine, Q. Interestingly, ProteinMPNN and Rosetta biases strongly disagree for lysine, K on the surface. Amino acid bias for ProteinMPNN is a function of the sampling temperature (see Figure S6) with low temperatures introducing more charged amino acids on the surface.

AlphaFold benchmarks

We ran all 5 AlphaFold ptm models with 3 recycles and selected the model with the highest average pLDDT as described in the AlphaFold paper (*9*) using only a single sequence as an input. For results described in Figure 2C and Figures S7, S8, S9, we generated 8 sequences per target backbone for 396 monomers (with maximum length of 300 residues) from the test set and used AlphaFold to predict structures for these sequences. Dependence on the inference noise level and sampling temperature for sequence recovery and relative AlphaFold success rate for the MPNN model trained with 0.2Å are shown in Figure S7A, B. Both of these metrics decrease with higher levels of noise and temperature. We also generated 8 sequences per target backbone for 277 homomers (with maximum length of 400 residues) and plotted sequence recovery and inter-chain predicted aligned error (PAE) as a function of the MPNN training noise level, Figure S7C. The trend is very much the same as for the monomer case showing that sequences from slightly noised MPNN models are more easily decoded by AlphaFold. It is important to notice that AlphaFold success rate for the single sequence prediction depends on the number of recycles used during the inference. We benchmarked 1, 2, 3, 6, and 12 recycles for the MPNN monomer sequences, see Figure S7D. The success rate monotonically increases suggesting that more powerful single sequence structure prediction models might have even higher success rates, i.e. MPNN sequences are correctly encoding structures, but it is hard to predict those structures using only single sequence information. Finally, we looked at the dependence between AlphaFold pLDDT and true lDDT-Ca, i.e. between native target backbone for MPNN and predicted AlphaFold backbone, Figure S8. The correlation is very much like in the original AlphaFold paper (*9*) in this single sequence regime with slight underestimation of lDDT-Ca.

Ca-only ProteinMPNN

We trained ProteinMPNN which used Ca coordinates only as an input instead of full backbone coordinates to have a way to generate sequences for coarse, or approximately correct backbones. In the similar way as for the full atom ProteinMPNN adding inter-atom distances as edge features helped to improve model's performance. We added distances between triplets of Ca atoms: Ca_{i-1}, Ca_i, Ca_i and Ca_{j-1}, Ca_j, Ca_j for residues i and j encoded as 16 Gaussian radial basis functions. The rest of the architecture is the same as for the full

atom version. Sequence recovery and relative AlphaFold success rate as a function of training noise level are shown in Figure S9.

Experimental methods

LM0878 (Figure 1D) was expressed in TBM-5052 medium with 50 μg/mL Kanamycin for 24 hours at 37°C with shaking at 225 rpm using the autoinduction method before harvesting via centrifugation at 4000xg for 5 minutes. Cells were lysed in 30ml of wash buffer (20 mM Tris, 300 mM NaCl, 25 mM Imidazole, pH 8.0) with sonication at 4°C. Lysates were then centrifuged for 45 minutes at 14000xg and applied to Ni-NTA resin that was pre-equilibrated with wash buffer. The resin was washed with 30 column volumes of wash buffer. 6xhis affinity tags were removed via on-bead SNAC (*26*) cleavage. Resin was washed with 20CV of cleavage buffer (100 mM CHES, 100 mM Acetone oxime, 100 mM NaCl, pH 8.6) prior to incubation with 20ml cleavage buffer and 2mM $NiCl_2$ overnight at room temperature. Flow through was collected and concentrated to 1ml using 3K protein concentrators (Millipore Sigma) before size exclusion chromatography using an S75 10/300 GL increase column (GE Healthcare).

LM0878 was crystallized through sitting drop vapour diffusion at room temperature in 0.1M citric acid pH 3.5 and 3M sodium chloride. Prior to harvesting and flash freezing in liquid nitrogen, crystals were transferred to the crystallization condition with 25% ethylene glycol. Diffraction data was collected at 100K at the Advanced Light Source beamline 8.2.1. Images were integrated using XDS 20220110 (*27*), with Aimless (*28*) used for scaling and merging. The design model was used as the search model for molecular replacement with Phaser 2.8. (*29*) Model building and refinement was done using Coot 0.9.8 (*30*), and Phenix refine from Phenix 1.20. (*31*) All structures were validated using MolProbity 4.5.1. (*32*) Crystallographic statistics are available in Table S1. Crystallographic data has been deposited to the protein bank with the PDB ID 8CYK.

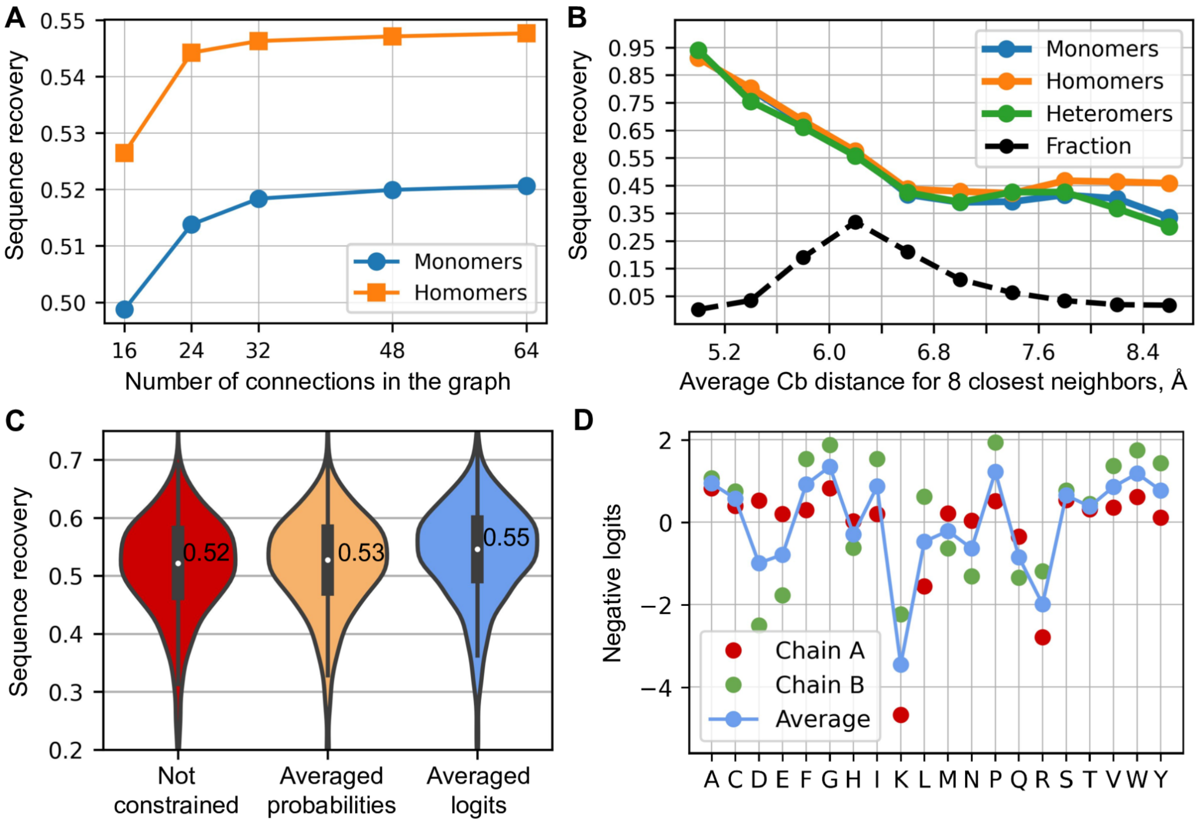

Fig. S1. In silico validation results. (**A**) Sequence recovery as a function of the number of nearest neighbors in the graph. (**B**) Sequence recovery as a function of burial for monomers, homomers, and heteromers. (**C**) Comparing three different ways of generating sequences for homomers: unconstrained (treating as non-symmetric), averaging predicted probabilities, averaging predicted logits. (**D**) An example showing negative logits predicted by the model for chain A and chain B in the homodimer. The blue curve shows averaged logits which will be normalized to sample an amino acid.

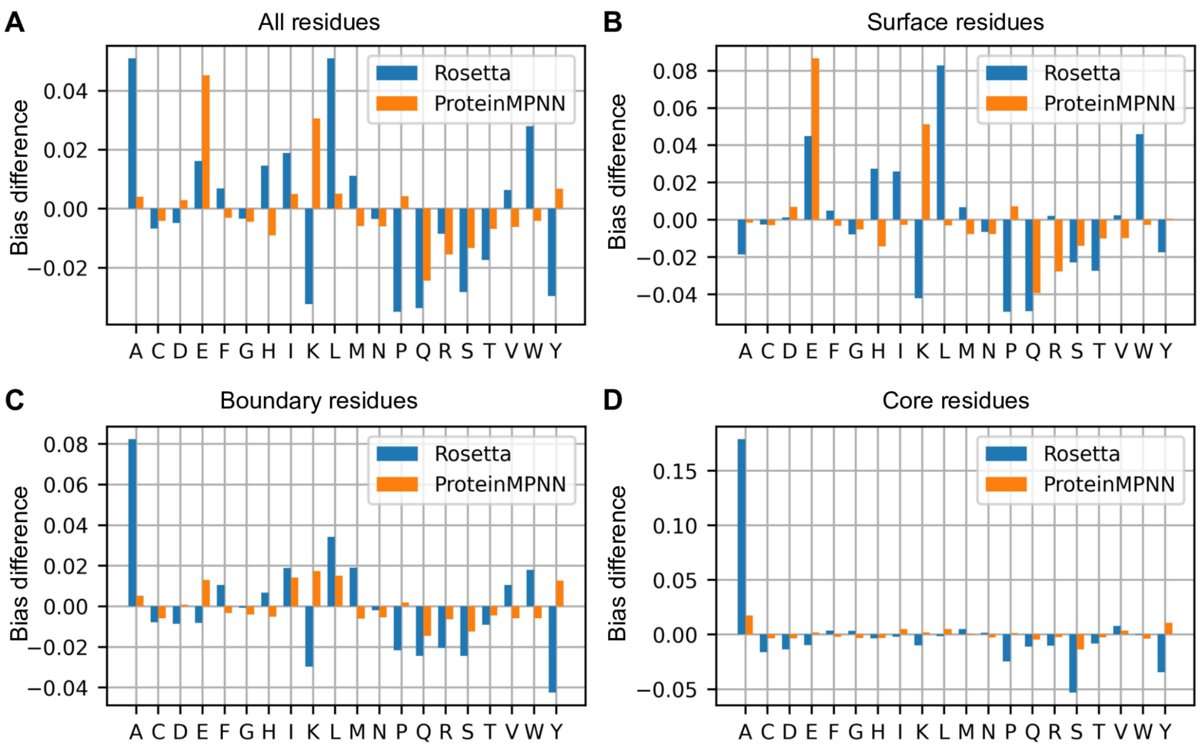

Fig. S2. Difference in compositional bias for Rosetta and ProteinMPNN. (**A**) Bias for all residues in the monomer chain. (**B**) Bias for surface residues. (**C**) Bias for boundary residues. (**D**) Bias for core residues.

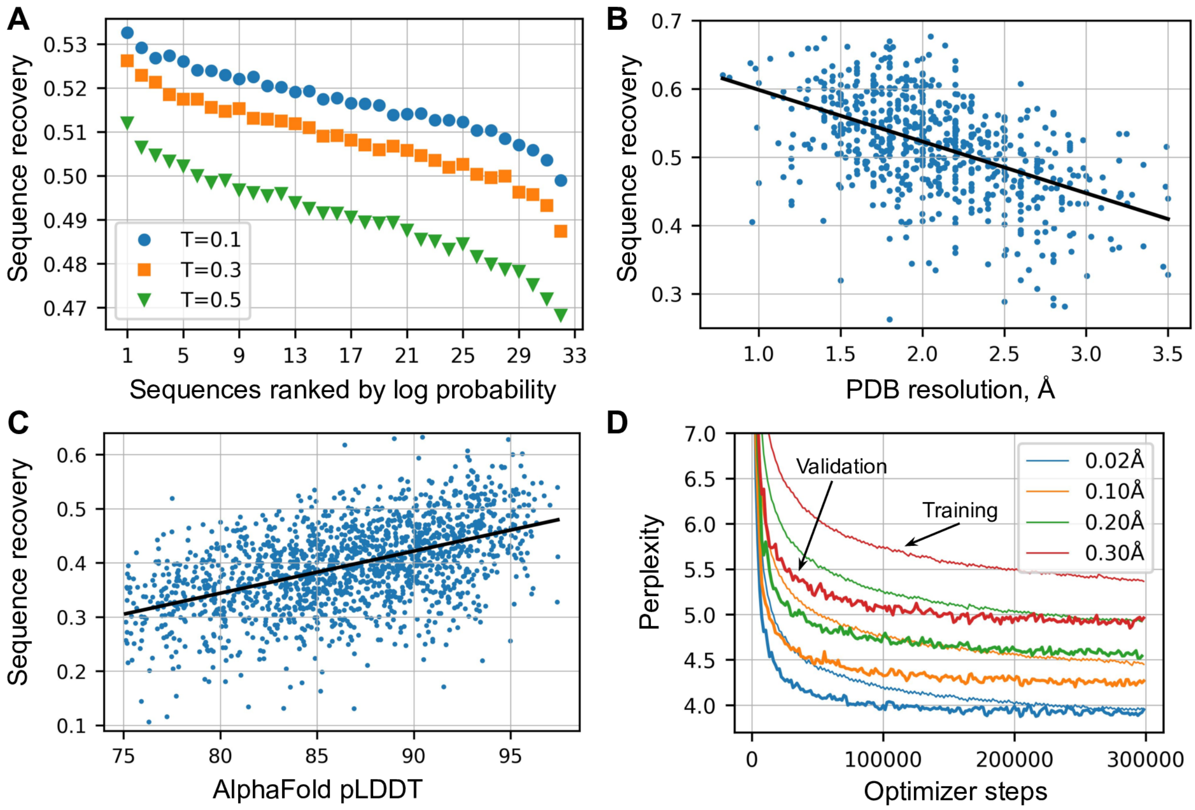

Fig. S3. Sequence recovery dependence on confidence and backbone quality. (**A**) We generated 32 sequences for every backbone in the test set of 690 PDB monomers using sampling temperatures 0.1, 0.3, 0.5 and ranked them using the log probability of the model (confidence). (**B**) Sequence recovery as a function of PDB resolution using 0.02Å noised MPNN model for a set of 690 PDB monomers. The black line shows a least squares linear fit. Spearman's rank correlation was -0.487. (**C**) Sequence recovery as a function of AlphaFold model confidence (average pLDDT) for a set of 1621 UniRef50 models. Spearman's rank correlation was 0.502. (**D**) Training and validation perplexities as a function of optimizer steps for different levels of backbone noise. Backbone noise and dropout (0.1) was not applied during the validation.

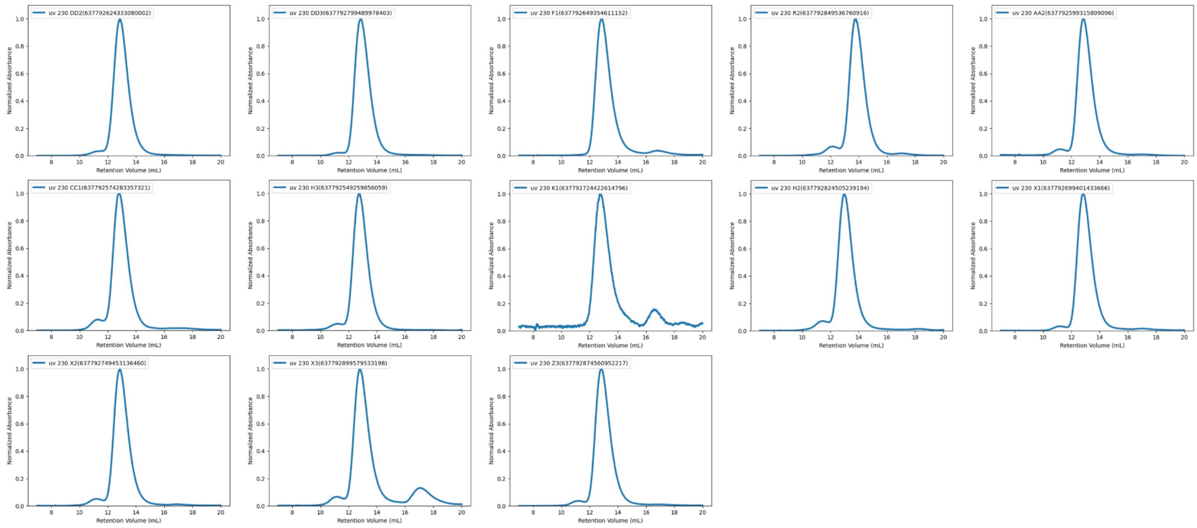

Fig. S4. **Size exclusion chromatography of proteinMPNN designed two-component tetrahedron nanoparticles.** 13/76 nanoparticle designs eluted at ~13 mL on a Superdex S200 10/300 Increase column, corresponding to nanoparticles of ~1 MDa. On the y-axis is normalized absorbance (uv 230), on the x-axis is retention volume [mL].

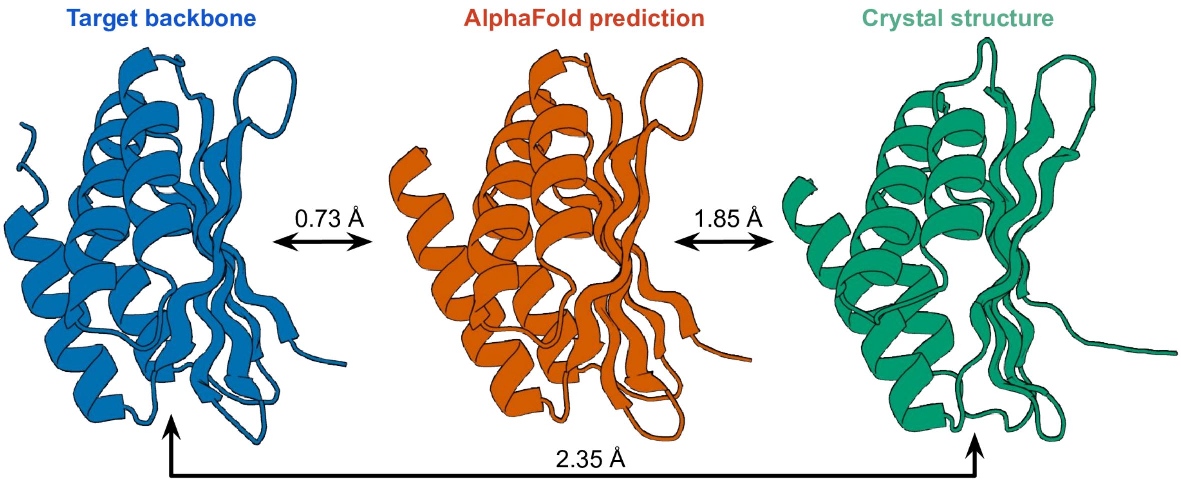

Fig. S5. Designed backbone and crystal structure comparison. Target backbone (LM0878) from hallucination on the left, AlphaFold prediction using ProteinMPNN sequence in the middle, and crystal structure on the right (deposited to PDB as 8CYK). Backbone RMSDs are shown for every pair of backbones.

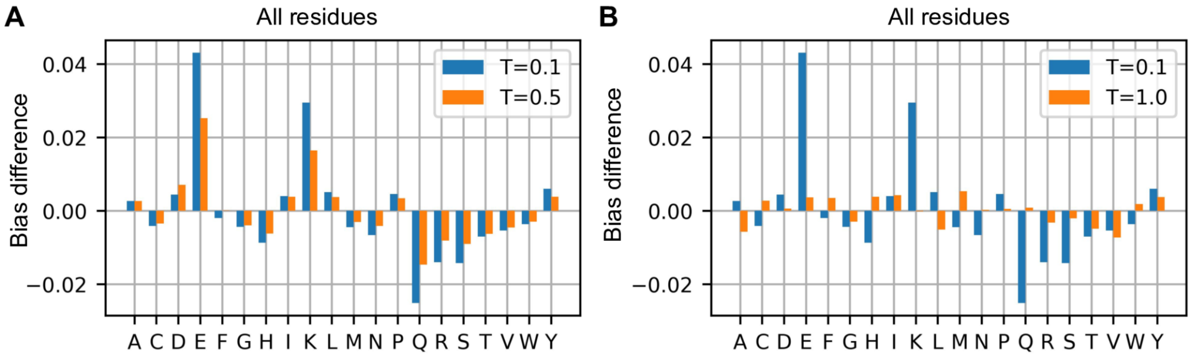

Fig. S6. ProteinMPNN bias for different sampling temperatures. (**A**) ProteinMPNN generates more charged amino acids in expense of the polar ones at low temperatures which likely leads to highly thermo-stable proteins. (**B**) Amino acid bias is very small at temperature T=1.0.

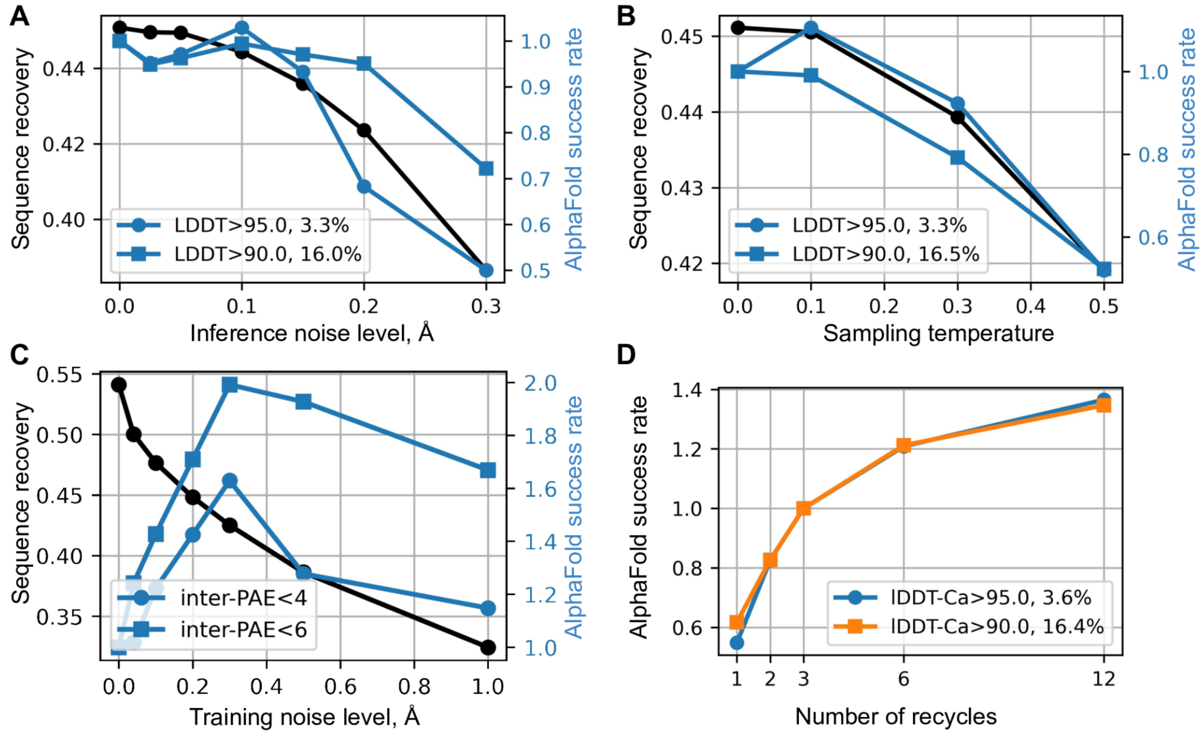

Fig. S7. Sequence recovery and AlphaFold (AF) benchmarks for the ProteinMPNN trained with 0.2Å noise level. (**A**) Sequence recovery and AF success rate for monomeric structures with true LDDT>95.0, 90.0 as a function of noise applied to backbones during the inference, 1.0 corresponds to 3.3% absolute rate for 95.0 LDDT cutoff and to 16.0% for 90.0 cutoff. (**B**) Same as A, but as a function of sequence sampling temperature. (**C**) Sequence recovery and AF success rate for homomers with inter-chain PAE<4.0, 6.0 as a function of training noise level, 1.0 corresponds to 2.4% for the 4.0 PAE cutoff, and to 5.6% for the 6.0 cutoff. (**D**) AlphaFold success rate as a function of number of AlphaFold recycles for monomers.

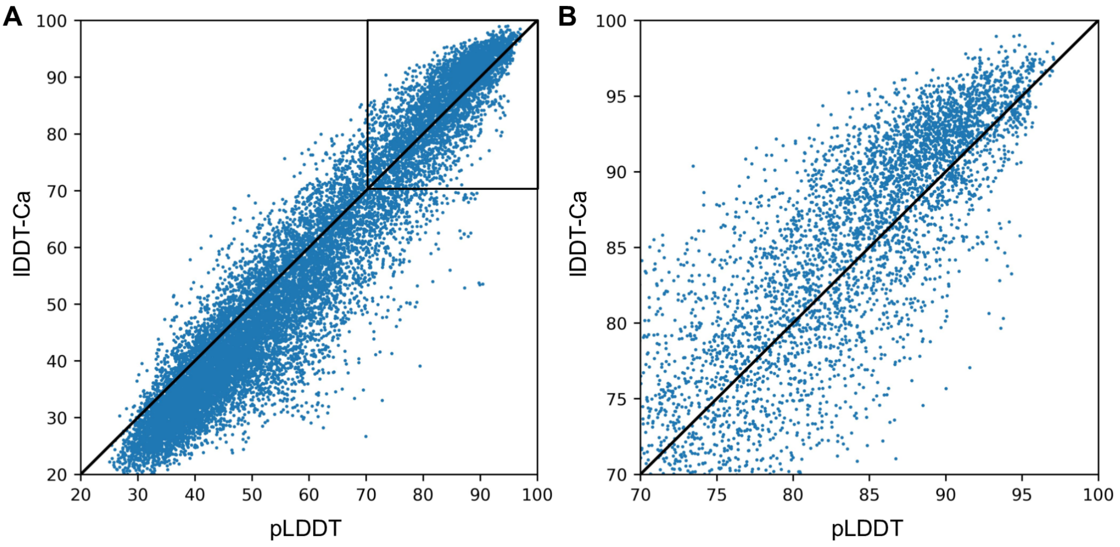

Fig. S8. Comparing true lDDT-Ca with AlphaFold predicted pLDDT for MPNN sequence redesigns on native PDB backbone monomers using single sequence prediction. lDDT-Ca is calculated between the native backbone input to the MPNN and the AlphaFold output using MPNN sequence. (**A**) pLDDT and lDDT-Ca are highly correlated in this regime. (**B**) Zoomed in version of A showing that true lDDT-Ca is slightly underestimated by pLDDT.

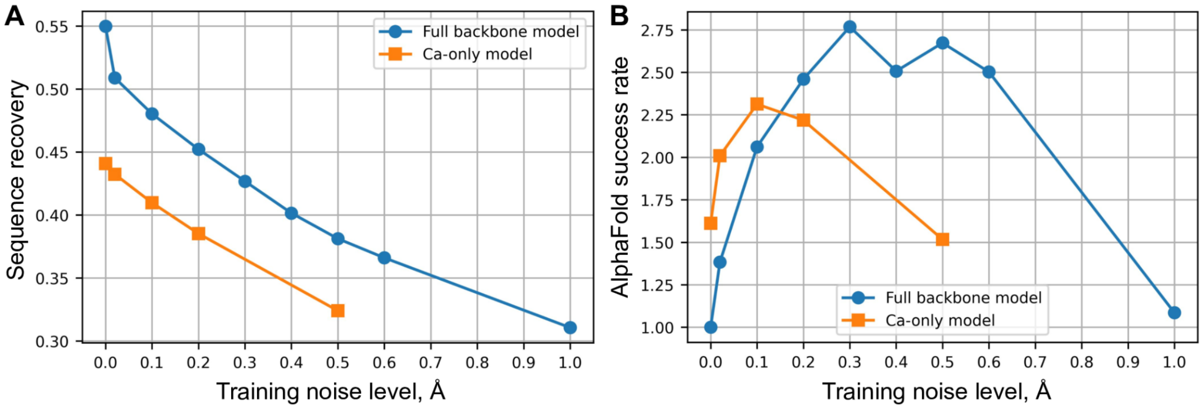

Fig. S9. Benchmarks of Ca-only ProteinMPNN model for monomers. (**A**) Sequence recovery as a function of training noise level for Ca-only and full backbone models. (**B**) Relative AlphaFold success rate as a function of training noise level for Ca-only and full backbone networks for the models with lDDT-Ca>90.0, 1.0 is equal to 6.7% success rate.

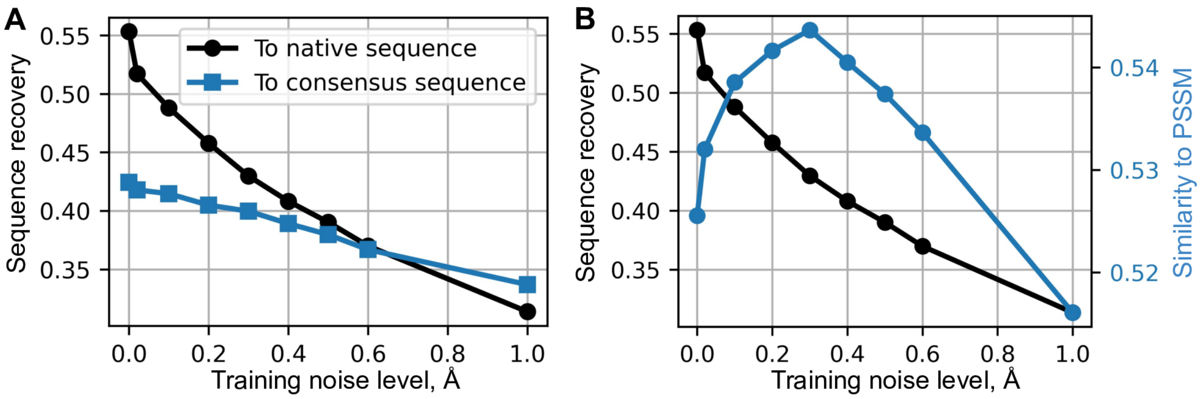

Fig. S10. Sequence comparison against consensus and PSSM distributions (**A**) Sequence recovery with respect to native and consensus sequences as a function of training noise level using temperature 0.1 (**B**) Sequence recovery and similarity to PSSM defined as 1-JS_divergence between PSSM distribution from multiple sequence alignment (details) and empirical ProteinMPNN distribution for 128 generated sequences.

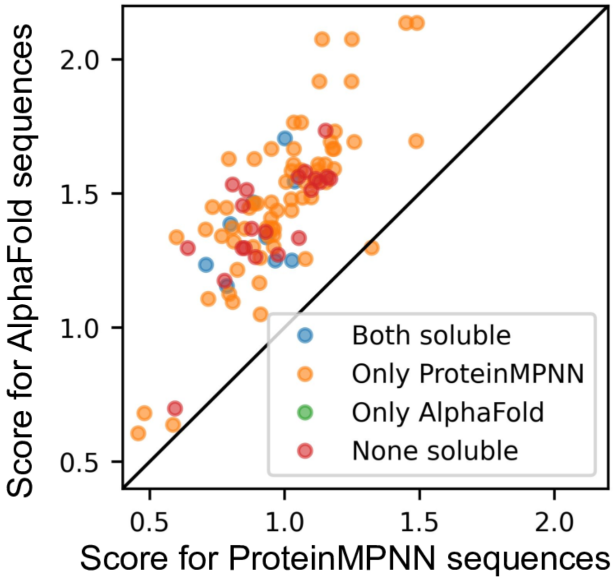

Fig. S11. Comparing ProteinMPNN scores (average negative log likelihoods) for AlphFold hallucinated and ProteinMPNN redesigned sequences for the same backbones. Colors indicate if expressed sequences were soluble from both methods, only from ProteinMPNN, only from AlphaFold, or none of them were soluble.
