# Supplementary material for: Robust deep learning based protein sequence design using ProteinMPNN
Source: Science. Author manuscript; Available in PMC 2023 Mar 9. (PMC9997061; doi:10.1126/science.add2187)
Supplement: Supp T1 [file NIHMS1874648-supplement-Supp_T1.pdf]

Supplementary Materials for

Robust deep learning based protein sequence design using ProteinMPNN

J. Dauparas[1,2], I. Anishchenko[1,2], N. Bennett[1,2,4], H. Bai[1,2], R. J. Ragotte[1,2], L. F. Milles[1,2], B. I. M. Wicky[1,2], A. Courbet[1,2,3], R. J. de Haas[6], N. Bethel[1,2,3], P. J. Y. Leung[1,2,4], T. F. Huddy[1,2], S. Pellock[1,2], D. Tischer[1,2], F. Chan[1,2], B. Koepnick[1,2], H. Nguyen[1,2], A. Kang[1,2], B. Sankaran[5], A. K. Bera[1,2], N. P. King[1,2], D. Baker[1,2,3]*

[1] Department of Biochemistry, University of Washington, Seattle, WA, USA.
[2] Institute for Protein Design, University of Washington, Seattle, WA, USA.
[3] Howard Hughes Medical Institute, University of Washington, Seattle, WA, USA.
[4] Molecular Engineering Graduate Program, University of Washington, Seattle, WA, USA
[5] Berkeley Center for Structural Biology, Molecular Biophysics and Integrated Bioimaging, Lawrence Berkeley Laboratory, 1 Cyclotron Road, Berkeley, CA 94720, USA.
[6] Department of Physical Chemistry and Soft Matter, Wageningen University and Research, Wageningen, The Netherlands.

Correspondence to: dabaker@uw.edu

This PDF file includes:

Tables S1 to S1

	**HALC1_878 (PDB ID: 8CYK)**
**Data Collection**	
Space group	I 1 2 1
Cell dimensions	
a, b, c (Å)	64.17, 55.45, 82.81
α, β, γ (°)	90.00, 94.00, 90.00
Resolution (Å)	48.98 - 1.65 (1.709 - 1.65)
Rmerge (%)	2.1 (19.4)
Rpim (%)	2.1 (19.4)
/σ(I)	15.91 (3.96)
CC 1/2	0.999 (0.942)
Completeness (%)	96.85 (95.70)
Redundancy	2.0 (2.0)
**Refinement**	
No. unique reflections	33946 (3322)
Rwork / Rfree (%)	18.5 (24.0) / 21.7 (28.1)
No. non-hydrogen atoms	2366
Macromolecules	2151
Water	215
Ramachandran favoured/allowed	98.83/1.17
**R.m.s. deviations**	
Bond lengths (Å)	0.011
Bond angles (°)	1.12
**B-factors (Å2)**	
Macromolecules	28.36
Solvent	39.25

Table S1. Crystallographic statistics for HALC1_878.
